# Supplementary material for: Novel Therapeutic Targets on the Horizon: An Analysis of Clinical Trials on Therapies for Bone Metastasis in Prostate Cancer
Source: Cancers (Basel). 2024 Jan 31;16(3):627. doi: 10.3390/cancers16030627 (PMC10854912; doi:10.3390/cancers16030627)
Supplement: Supplementary file 1 [file cancers-16-00627-s001.zip › cancers-2714324-supplementary.pdf]

Table S1. Clinical Trails failing in the treatment of bone metastases in prostate cancer

Trail	OS	Time to first SRE
TROG 03.04 RADAR [1]	No significant difference	N/A
CALGB 90202 [2]	No significant difference	No significant difference
ZEUS [3]	No significant difference	No significant difference
Hoskin et al. [4]	No significant difference	N/A
Wang F et al. [5]	Benefit	No significant difference
TRAPEZE [6]	Benefit	No significant difference
ZAPCA [7]	Benefit	No significant difference

1. Denham, J.W.; Joseph, D.; Lamb, D.S.; Spry, N.A.; Duchesne, G.; Matthews, J.; Atkinson, C.; Tai, K.H.; Christie, D.; Kenny, L.; et al. Short-term androgen suppression and radiotherapy versus intermediate-term androgen suppression and radiotherapy, with or without zoledronic acid, in men with locally advanced prostate cancer (TROG 03.04 RADAR): 10-year results from a randomised, phase 3, factorial trial. *Lancet Oncol* **2019**, *20*, 267-281, doi:10.1016/s1470-2045(18)30757-5.
2. Smith, M.R.; Halabi, S.; Ryan, C.J.; Hussain, A.; Vogelzang, N.; Stadler, W.; Hauke, R.J.; Monk, J.P.; Saylor, P.; Bhoopalam, N.; et al. Randomized controlled trial of early zoledronic acid in men with castration-sensitive prostate cancer and bone metastases: results of CALGB 90202 (alliance). *J Clin Oncol* **2014**, *32*, 1143-1150, doi:10.1200/jco.2013.51.6500.
3. Wirth, M.; Tammela, T.; Cicalese, V.; Gomez Veiga, F.; Delaere, K.; Miller, K.; Tubaro, A.; Schulze, M.; Debruyne, F.; Huland, H.; et al. Prevention of bone metastases in patients with high-risk nonmetastatic prostate cancer treated with zoledronic acid: efficacy and safety results of the Zometa European Study (ZEUS). *Eur Urol* **2015**, *67*, 482-491, doi:10.1016/j.eururo.2014.02.014.
4. Hoskin, P.; Sundar, S.; Reczko, K.; Forsyth, S.; Mithal, N.; Sizer, B.; Bloomfield, D.; Upadhyay, S.; Wilson, P.; Kirkwood, A.; et al. A Multicenter Randomized Trial of Ibandronate Compared With Single-Dose Radiotherapy for Localized Metastatic Bone Pain in Prostate Cancer. *J Natl Cancer Inst* **2015**, *107*, doi:10.1093/jnci/djv197.
5. Wang, F.; Chen, W.; Chen, H.; Mo, L.; Jin, H.; Yu, Z.; Li, C.; Liu, Q.; Duan, F.; Weng, Z. Comparison between zoledronic acid and clodronate in the treatment of prostate cancer patients with bone metastases. *Med Oncol* **2013**, *30*, 657, doi:10.1007/s12032-013-0657-x.
6. James, N.D.; Pirrie, S.J.; Pope, A.M.; Barton, D.; Andronis, L.; Goranitis, I.; Collins, S.; Daunton, A.; McLaren, D.; O'Sullivan, J.; et al. Clinical Outcomes and Survival Following Treatment of Metastatic Castrate-Refractory Prostate Cancer With Docetaxel Alone or With Strontium-89, Zoledronic Acid, or Both: The TRAPEZE Randomized Clinical Trial. *JAMA Oncol* **2016**, *2*, 493-499, doi:10.1001/jamaoncol.2015.5570.
7. Kamba, T.; Kamoto, T.; Maruo, S.; Kikuchi, T.; Shimizu, Y.; Namiki, S.; Fujimoto, K.; Kawanishi, H.; Sato, F.; Narita, S.; et al. A phase III multicenter, randomized, controlled study of combined androgen blockade with versus without zoledronic acid in prostate

cancer patients with metastatic bone disease: results of the ZAPCA trial. *Int J Clin Oncol* **2017**, *22*, 166-173, doi:10.1007/s10147-016-1037-2.
